# The placebo effect in allergen-specific immunotherapy trials

**DOI:** 10.1186/2045-7022-3-42

**Published:** 2013-12-21

**Authors:** Annemie Narkus, Ulrike Lehnigk, Dietrich Haefner, Regine Klinger, Oliver Pfaar, Margitta Worm

**Affiliations:** 1 Medical Department, Allergopharma GmbH & Co KG, Reinbek, Germany; 2 Department of Clinical Psychology, Behaviour Therapy, University of Hamburg, Hamburg, Germany; 3 Department of Otorhinolaryngology, Head and Neck Surgery, University Hospital Mannheim, Mannheim, Germany; 4 Allergy-Center-Charité, Department of Dermatology and Allergy, Charité-Campus Mitte, Universitätsmedizin, Berlin, Germany

**Keywords:** Allergen-specific immunotherapy, Allergen exposure, Placebo-controlled studies, Placebo effect

## Abstract

**Background:**

Double-blind, placebo-controlled (DBPC) trials are the gold standard for demonstrating clinical efficacy and tolerability. The placebo effect, although an important feature in placebo-controlled studies, has never been systematically investigated in allergen-specific immunotherapy (SIT) studies. This study was performed to examine the placebo response in SIT trials that employed a baseline observational period and two treatment years using a symptom-medication-score (SMS) as the primary endpoint.

**Methods:**

The placebo effect was evaluated in six DBPC SIT studies (five studies using subcutaneous SIT (SCIT) and one sublingual (SLIT)), two grass, two birch and two house dust mite (HDM) SIT, including a total of 472 adult patients treated with a placebo. The results were reported as changes from baseline of the SMS area under the curve after two years of perennial placebo therapy during the respective evaluation periods. Pollen counts and IgG_4_ levels were additionally analysed.

**Results:**

Subcutaneously treated placebo patients displayed a marked decrease in the SMS. The mean placebo effect in the SCIT trials with comparable allergen exposure was up to 41% in the second treatment year and, in contrast, reached only 1% in the SLIT trial. Allergen exposure had an inverse influence on the placebo effect. No changes from baseline in allergen specific IgG_4_ antibodies were observed in the placebo-treated patients.

**Conclusions:**

SIT studies display a significant placebo effect, mainly observed in subcutaneous immunotherapy, with high variability depending on the route of application and allergen exposure. Our findings indicate the differential role of the placebo effect in SIT efficacy depending on the route of administration and pollen exposure.

## Background

Double-blind, randomised controlled trials provide the highest level of evidence in clinical studies [1]. The major purpose of control groups is to discriminate between experimental group outcomes caused by the active treatment and those that are related to other factors, such as natural progression of the disease, observer or patient expectations or other treatments [2]. Clinical studies can use different types of controls [2], but despite some ethical issues [3], the use of placebos is considered the gold standard to demonstrate clinical efficacy and tolerability for many medicinal products [4].

The placebo design is said to control for all of the potential influences on the course of a disease (other than those arising from the pharmacologically active treatment). These features include spontaneous improvement (i.e., the natural history of the disease and regression to the mean), subject or investigator expectations, effect of being in a trial, use of other therapies and subjective elements of the diagnosis or assessment. The outcome differences between the pharmacologically active treatment and placebo groups define the treatment effect under trial conditions [5,6].

Allergic diseases are characterised by variable clinical responses because of the unpredictability and variability of allergen exposure and, more importantly, the subjective nature of symptom assessment [7]. Accordingly, double-blind, placebo-controlled trials (DBPC) are recommended by international guidelines for studies investigating the efficacy and safety of allergen-specific immunotherapy (SIT) [8].

The placebo effect is an interesting feature in placebo-controlled studies and refers to the improvement observed in a percentage of patients treated with placebos in a wide range of clinical conditions, either in trials or in clinical practice [9]. It is a genuine psychobiological event attributable to the overall therapeutic context [3]. Psychologically, it is attributed to classical conditioning mechanisms and patient expectations [3,6,10]. Several studies have examined the placebo effect in DBPC studies in trials comparing the placebo with no treatment in different diseases and for different types of outcomes [10-14]. The impact of placebo effects has been described in many reviews [6]. High rates of placebo efficacy were attained in a study with patients with atopic dermatitis [10]. Currently, the placebo effect is discussed in matters of clinical relevance [15]. Because placebos can actually produce results comparable to specific drugs without deception [10,16], it is important to exploit the placebo effect by applying its mechanisms [17] and thus deliberately boosting the efficacy of treatments.

The placebo effect has been analysed in different pharmacologic and placebo treatments in allergic diseases [7,10,18] but never in placebo-controlled SIT clinical trials. In SIT trials, treatment efficacy is usually expressed as the difference between the symptom-medication-score (SMS) in the active treatment group and the placebo group after treatment. A proper evaluation of the placebo effect in SIT trials requires a baseline period to document the effect of exposure and serves as a reference value for calculating changes. SIT trials with a baseline season are limited because they are deemed to be costly and time-consuming [8].

The aim of this study was to examine the placebo effect in different SIT trials with comparable designs consisting of one observational season (baseline) followed by two treatment years using SMS as the efficacy parameter. Our data highlight the characteristics and clinical relevance of the placebo response and provide relevant information for designing and interpreting future clinical studies in allergen-specific immunotherapy.

## Methods

### Study characteristics

To avoid the well-known heterogeneity of SIT studies and to include in our analysis only comparable trials, we considered in the present analysis all DBPC phase III SIT studies of the company (Allergopharma) that were comparable and homogeneous for all of the following parameters (n = 6): a prospective baseline observation period (a baseline season in the case of pollen studies); the same inclusion and exclusion criteria, as previously recommended [19]; the same baseline patient clinical characteristics (adult patients with allergic rhinitis (AR) with or without bronchial asthma, GINA I and II) [20]; a two-year treatment period; the same evaluation method for allergen exposure; the same length of treatment observation period; a validated method to assess the SMS [21]; the same method for immunological measurements; and the same components and appearance of placebo treatments.

Two studies included patients suffering from a grass pollen allergy, two included patients with a birch pollen allergy and two included patients with a house dust mite (HDM) allergy (Table 1). Five studies were performed with subcutaneous immunotherapy (SCIT), and one used a sublingual extract (SLIT). Three of these studies have been published before [22-25]; data for the remaining three (SCIT HDM 2, SCIT Grasses and SCIT Birch 2) have not yet been published.

**Table 1:** Characteristics of the evaluated studies and included patients (FAS)

**Extract**	**Histamine**	**No. of placebo patients**	**Age (year, mean,** ±**SD)**	**Gender (M/F)**
SCIT HDM 1 (22)	+	62	28.6 (±9.8)	36/26
SCIT HDM 2 *	+	50	32.0 (±10.2)	25/25
SCIT Grasses *	+	87	31.7 (±9.4)	52/35
SLIT Grasses (23,25)	-	55	34.1 (±10.3)	32/23
SCIT Birch 1 (24)	-	98	38.4 (±11.4)	45/53
SCIT Birch 2 *	-	120	39.0 (±11.2)	58/62

Legend: *data on file.

For all of the studies ethical and regulatory approval was obtained from the local ethics committee(s) and the local health authorities in the respective countries (United Kingdom, Poland, Germany, Italy, Macedonia, Sweden, and Finland). Written informed consent was obtained from the patient for each of the studies prior to any study related procedure.

The placebo treatment contained all of the constituents of the active product, except for the allergens. In case of SCIT, the placebo contained aluminium-hydroxide, and in three studies, it contained histamine (0.125 mg/ml in the highest strength) for blinding.

### Assessment

The results were reported as changes from the baseline of the area under the curve (AUC) of SMS after 2 years of perennial placebo therapy (mean values). The SMS of all of the included studies was based on the validated Allergy Control Score™ [21]. The evaluation periods covered 21 days for birch pollen, 42 days for grass pollen and 28 days for HDM allergies. To compare the subjective and objective outcomes in the placebo group, IgG_4_ changes in the placebo group were also evaluated.

### Assessment of allergen exposure

Because different pollen exposure levels in different seasons can influence the results, the daily mean AUCs of birch and grass pollen (grains/m^3^) were assessed during the evaluation periods (https://ean.polleninfo.eu/Ean/). Placebo patients were paired with an allocated pollen trap. The maximum distance between trial centres and pollen stations was 100 km. If one trap provided no results, the closest station with results was chosen for the assessment. Pollen levels were analysed using the median AUC of the pollen count during the assessment period. This period began 10 or 7 days before the pollen peak day and ended 31 days or 13 days after the peak day for grass or birch pollen, respectively.

For patients with AR triggered by HDM, a positive HDM exposure with a semi-quantitative biochemical test (Acarex®, Davimed Pharma + HealthCare GmbH, Germany ) was performed before and 2 years after therapy to ensure comparable exposure during the baseline and evaluation periods after 2 treatment years.

### IgG_4_ evaluation

In all studies, IgG_4_ measurements were performed by the same laboratory using the method described previously [26].

## Results

The six studies included 472 placebo-treated patients (248 males and 224 females; mean age 34 ± 10.4 years) (Table 1). A total of 87 patients were treated with SCIT grass pollen placebo, 55 patients were treated with SLIT grass pollen placebo, 218 patients were treated with SCIT birch pollen placebo and 112 patients were treated with SCIT HDM placebo.

**Figure 1:**
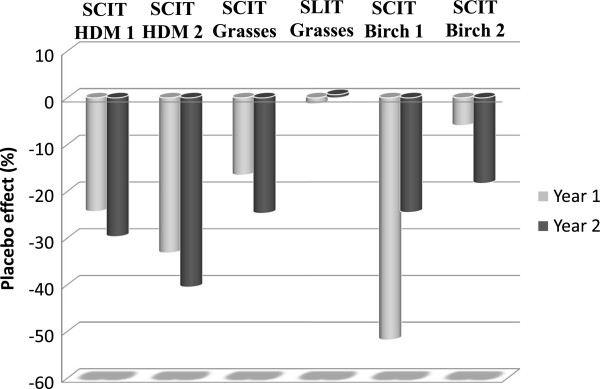
Mean% change from baseline for the AUC of SMS in the different studies and treatment years.

**Figure 2:**
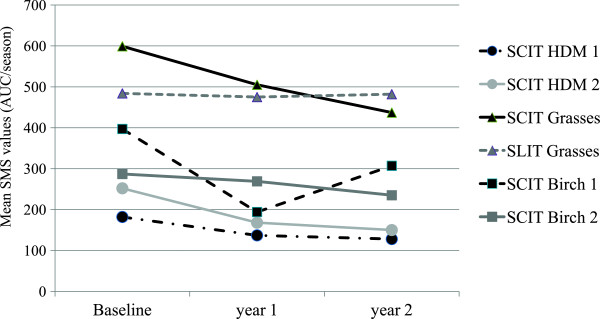
**Mean AUC of SMS at baseline after the first and the second treatment year in the placebo groups of the different trials.** Legend: the SDs are as follows: SCIT HDM 1 (baseline ±104, first year ±112, second year ±109), SCIT HDM 2 (baseline ±122, first year ±110, second year ±104), SCIT grasses (baseline ±295, first year ±294, second year ±305), SLIT grasses (baseline ±218, first year ±321, second year ±300), SCIT birch 1 (baseline ±156, first year ±133, second year ±168), SCIT birch 2 (baseline ±131, first year ±162, second year ±168).

A marked SMS decrease in placebo-treated patients was detectable in all but the SLIT trial. Table 2 shows the baseline values for AUC of SMS in these trials and the changes from baseline after the first and second treatment year. Figure 1 displays the mean% change from baseline for the AUC of SMS in the different studies and treatment years, whereas Figure 2 shows the mean AUC SMS values in the different studies at baseline and during the evaluation periods at one and two treatment years. Patients allergic to pollen showed higher mean daily SMS values at baseline (14.3/11.5 in the grass trials and 18.9/13.7 in the birch trials) compared to the patients allergic to HDMs (6.5/9).

**Table 2:** Changes from baseline as well as changes in the per cent of the AUC of SMS in the placebo group during the studies

**Extract**	**SMS at baseline**	**SMS mean change from baseline**	**SMS mean change from baseline**	Placebo effect SMS %*	
	**(mean)**	**1st yr**	**2nd yr**	**1st year**	**2nd year**
**SCIT HDM 1**	181.7	−44.2	−53.9	**−24.3**	**−29.7**
**SCIT HDM 2**	251.7	−83.7	−102.0	**−33.2**	**−40.5**
**SCIT grasses**	599.3	−98.8	−148.3	**−16.5**	**−24.7**
**SLIT grasses**	484.0	−6.2	+3.6	**−1.3**	**+0.7**
**SCIT Birch 1**	396.9	−205.5	−97.4	**−51.8**	**−24.5**
**SCIT Birch 2**	286.8	−16.8	−52.4	**−5.9**	**−18.3**

Legend: *calculated as change/baseline (in%).

**Figure 3:**
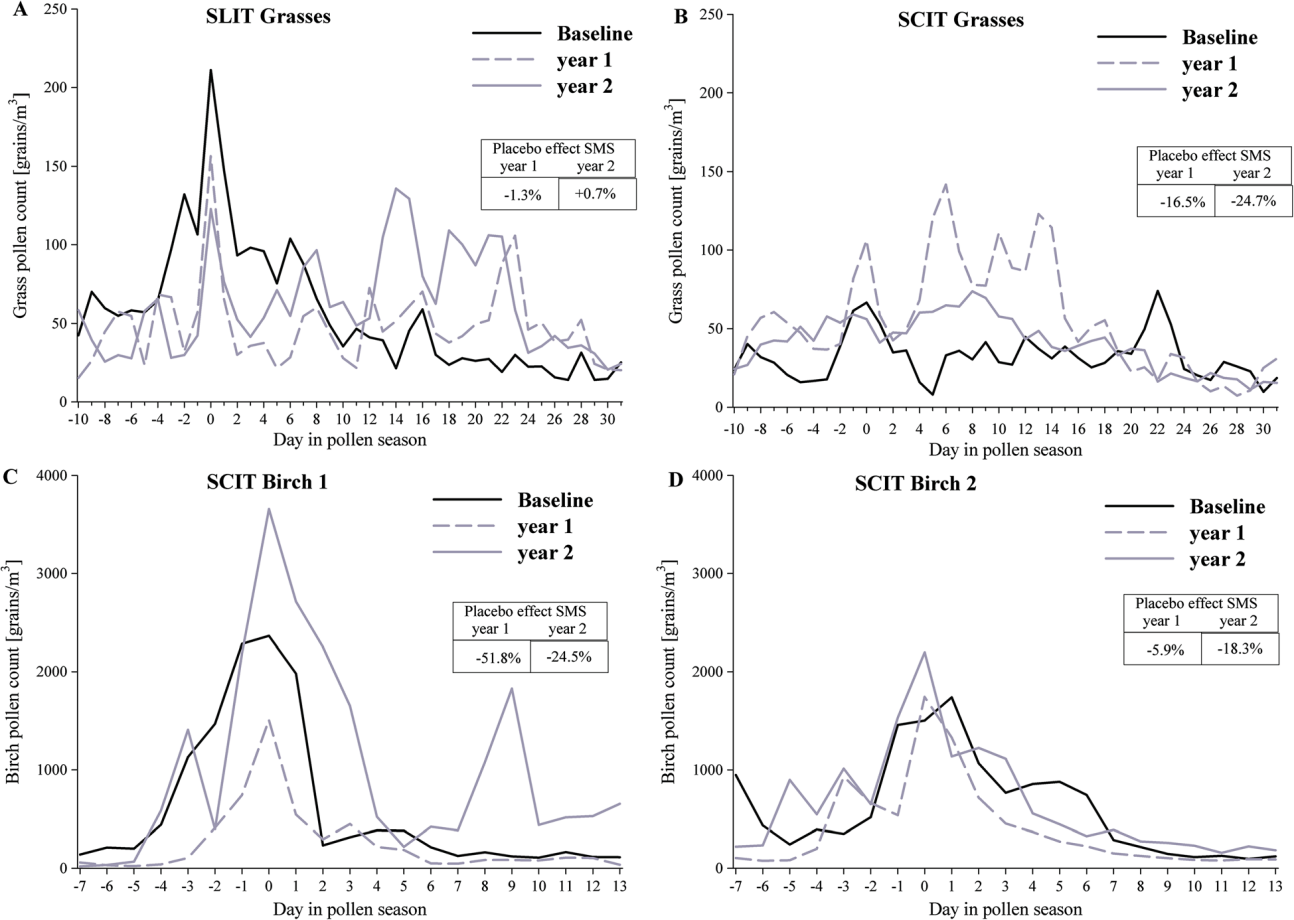
**Daily mean pollen count during baseline and the first and second treatment years in the different pollen trials.** Legend: **A** SLIT grasses, **B** SCIT grasses, **C** birch trial 1, **D** birch trial 2.

These data must be matched for the different allergen exposures faced by placebo patients in the different studies and evaluation periods. Table 2 shows the placebo effect in the HDM allergic patients who had a comparable HDM allergen exposure at baseline and at the first and second treatment years. Figure 3 displays the daily mean pollen exposure at baseline and during the pollen seasons after one and two treatment years for grass or birch pollen studies. Grass pollen exposure was similar throughout the study periods in the SLIT trial and resulted in −1.3% and +0.7% mean changes in AUC SMS (Figure 3A). In the SCIT trial, the grass pollen exposure was much higher during the first season and resulted in a placebo effect of 16.5%; pollen exposure decreased in the second pollen season but was still enhanced compared to baseline resulting in a placebo effect of −24.7% (Figure 3B). The pollen exposure in Birch trial 1 showed even higher fluctuations, with reduced exposure in the first treatment year that resulted in a placebo effect of 51.8%, which was followed by an increase in exposure in the second year compared to baseline (a 24.5% placebo effect) (Figure 3C). Birch pollen exposure was constant in Birch Trial 2. The placebo effect was only 5.9% in the first treatment year and increased to 18.3% in the second year (Figure 3D).

For five of the six trials, IgG_4_ values, which were evaluated using a comparable method, were available. Contrary to the clinical data, the IgG_4_ levels did not change in placebo patients during the two years of treatment.

## Discussion

The present investigation is the first analysis of the placebo response in adult allergic patients enrolled in SIT trials with homogeneous study designs, comparing this response in different allergens and different routes of administration. All of the studies covered a two-year treatment period after a baseline observation phase and used a similar tool to assess efficacy after treatment. Our data showed a high placebo effect in SIT, which was much less prominent in SLIT than in SCIT.

The nervous system and psychological effects are important features of allergic and immunological reactions [7], both in asthma and AR [27,28]. Allergic diseases constitute a group that is highly susceptible to placebo effects, and this effect has been evaluated in placebo-controlled studies of anti-allergic drugs [18].

A meta-analysis of the efficacy of different drug treatments authorised in the United States for allergic rhinitis showed a 15% improvement in the total symptom score (TSS) for seasonal AR and a 24.8% improvement for perennial AR in placebo-treated patients [29]. Similar placebo effects have been detected in a meta-analysis evaluating the efficacy of antihistamines, inhaled steroids and a long-acting β-agonist in AR, allergic asthma and atopic dermatitis; the analysis observed an average placebo effect of 23.0% [7]. A subsequent study investigating the efficacy of different antihistamines compared to placebos in patients with AR found no significant difference between the treatment groups. The physicians’ assessments showed 44% and 40% overall treatment success rates for the antihistamines fexofenadine HCl and loratadine, respectively, and a 36% treatment success rate in the placebo group. The patients’ assessments showed treatment success rates of 47%, 42% and 37%, for fexofenadine HCl, loratadine and a placebo, respectively [30].

It is well known that subjective endpoints (e.g., patient-reported outcomes and observer-reported outcomes involving patient cooperation) are more susceptible to placebo effects than objective variables [9,13,31]. This finding was also confirmed by our analysis; despite the marked placebo effect in SMS, no change from baseline in allergen specific IgG_4_ antibodies was observed in the placebo groups.

It has also been reported that the exposure route plays an important role in the magnitude of the placebo effect and that injections cause greater placebo effects than tablets or oral applications [32,33]. An increased placebo response was observed in the five studies using subcutaneous therapy compared with the SLIT study (max. 40.5% for SCIT versus 1.3% for SLIT in studies with comparable allergen exposure), which confirms that the administration route may result in different magnitudes of placebo effect in SIT trials. Although we could only include one SLIT trial, other trials support our results, both in studies with comparable pollen counts and in those with comparable mite exposures [34-36]. Ott et al. (35) showed no effect or a worsening in the SLIT placebo group during 3 years of treatment, and a placebo effect could only be observed in the follow-up period due to lower pollen exposure. The very low placebo effect in the SLIT studies is in contrast to the average placebo effect of 23% for pharmacotherapy of allergic diseases, as shown in a US meta-analysis [7]. One reason for this may be the long duration of SIT trials in contrast to studies with pharmacotherapy where patients will realise the burden of their disease over time. The contribution of the lower SMS in the SLIT versus the SCIT grass trial to the low placebo effect in the SLIT trial seems to be unlikely because even in the other SCIT trials with lower SMS values at baseline, a high placebo effect could be observed. Previous SCIT publications have also reported placebo effects similar to our study. Blumberga et al. [37] found 25% and 42% decreases in inhaled steroid use in placebo patients after 2 and 3 years, respectively. Varney et al. [38] observed a 32% reduction in symptom scores after 1 year of placebo SCIT treatment for patients with mite allergies. Walker et al. reported reductions of 15% in symptom scores and 18% in medication scores in placebo-treated-patients with grass allergies, although the pollen exposure was higher in the observation period after the treatment [39]. We could not include these SLIT and SCIT studies in our analysis due to the high heterogeneity of study designs and methods. Furthermore, we excluded studies in children or using pollen chambers because this would have added further complexity.

In addition to the administration route, one reason for the high placebo effects in SCIT may be that, according to clinical guidelines, the placebo must have the exact same composition as the active treatment, except for the allergen. The placebo in the analysed SCIT trials contained aluminium hydroxide and occasionally histamine for blinding. The known adjuvant effect of aluminium hydroxide may have contributed to the placebo effect in SCIT. Furthermore, the high placebo effect in the SCIT trials was a good indication that the blinding was effective in these clinical trials.

A highly important but simple reason for the high placebo effect in SCIT may be the fact that patients are taken care of regularly; they have to come to their physician to receive the injections; the patients and physicians have regular discussions about the disease and treatment, and exacerbations and other issues can be handled immediately. Consequently, patients will generally consider the burden of their disease less severe. The SLIT treatment is home-based, and the patients are left on their own without health care support and again will most likely have a minor placebo effect. However in a study setting, contact with physicians is normally still higher than in daily practice.

The DBPC studies analysed here reported in a prospective manner a baseline period monitored with the same outcomes, methods and scores used for post-treatment evaluation. Unfortunately, the majority of SIT studies do not include a baseline observation. Therefore, they can only assess a final difference between active- and placebo-treated patients but not real improvements or the placebo response. Some pollen trials assess baseline values retrospectively, but this method is not suitable because of many biases, the most important being a memory bias [40]. Official documents now recommend the inclusion of a prospective baseline period [41], although the World Allergy Organization states that although the inclusion of a baseline observation is correct in principle, it is not mandatory because it is “expensive and time consuming” [8].

Placebo patients who are allergic to pollen showed higher SMS values compared to patients that are allergic to HDM, most likely because of a reduced awareness of symptoms in perennial exposure. However, even this reduced “room for improvement” resulted in a high placebo effect that was up to 40.5%.

To evaluate placebo effects, the amount of exposure is critical. Our analysis showed that in HDM-allergic patients with comparable exposures between the baseline and the end of the treatment period, the placebo effect in SCIT ranged from 24.3% to 29.7% in study 1 and from 33.2% to 40.5% in study 2. With increased exposure, a reduced placebo effect resembling the SCIT grass trial (16.5% and 24.7%) or the second pollen season in Birch trial 1 (24.5%) was observed. In contrast, a low pollen exposure resulted in a high placebo effect of up to 51.8% (first year of Birch trial 1). The only exception seemed to be the low placebo effect in the first year of Birch trial 2, although no clear reason was apparent. We have analysed the relationship between changes in the SMS and pollen count and found statistically significant correlations for all studies and all treatment years. Thus, it can be concluded that the amount of allergen exposure is causally related to the magnitude of the placebo effect.

The high placebo effect observed in this SIT analysis must not be ignored and obviously needs further research on the placebo effectiveness in the clinical context of immunotherapy targeting the underlying mechanisms of this genuine psychobiological event. Classical conditioning as well as expectations induced via instructions enable the release of endogenous neurotransmitters, which imitate the expected or conditioned pharmacological effects in the sense of placebo effects [3,42,43]. In a sample of patients with atopic dermatitis, we previously showed that an analgesic placebo effect was also established via expectations induced by both mechanisms, i.e., classical conditioning and instructions; however, compared to the healthy control group in the patient group, the effect was maintained only in the groups who experienced classical conditioning [10]. This means that the experience of symptom relief is necessary to learn the placebo effect. Benedetti and colleagues [44] showed that analgesics always consist of two components, a pharmacological and a psychological (placebo) component. They proved this assumption within an alternative model of the double-blind, randomised controlled trials, namely, the “open-hidden paradigm”. Here, identical concentrations of active drugs are administered by a physician in an appreciable, e.g., visible (open condition) or hidden, manner (drug given by a computer), in which the patient is unaware of the timing of medication administration. So it is possible to dissociate the pure pharmacological effect of the treatment (hidden treatment) from the additional benefit of the psychological context resulting from knowing that the treatment is being administered. The difference between the outcomes following the administration of the expected and unexpected therapy can be seen as the placebo response or psychological component, even though no placebo treatment has been used. These findings require exploitation of the placebo effect in pharmacological treatment as much as possible. Placebo efficacy can be seen as a psychological add-on to the pharmacological component of a medication.

## Conclusions

In conclusion, we detected a placebo effect in SCIT trials from 24% to 41% in mite and from 6% to 25% in pollen allergic patients with comparable allergen exposure and up to 51.8% with reduced exposure, which is in contrast to 1.3% or less in the SLIT trial. The differences between the SCIT and SLIT trials may be explained by the treatment method (i.e., injections versus oral applications) or the ingredients, such as aluminium hydroxide or histamine, included in the placebos in the SCIT trials in order to meet regulatory requirements or to maintain blinding.

This observation underscores the value of a proper baseline observation period in SIT placebo-controlled trials.

## Abbreviations

AR: Allergic rhinitis; AUC: Area under the curve; DBPC: Double-blind, placebo-controlled; GINA: Global initiative for asthma; HDM: House dust mites; IgG4: Immunoglobulin G, isotype 4; SCIT: Subcutaneous allergen-specific immunotherapy; SIT: allergen-specific immunotherapy; SLIT: Sublingual allergen-specific immunotherapy; SMS: Symptom-medication-score.

## Competing interests

A. Narkus, U. Lehnigk and D. Haefner are employees of Allergopharma GmbH and Co KG, Reinbek Germany and the studies were performed by Allergopharma GmbH and Co KG.

RK, OP and MW declare that they have no competing interests.

## Authors’ contributions

AN accounted for the design of the studies and the investigation and drafted the manuscript; UL participated in the design of the investigation as well as the preparation and interpretation of the data; DH was in charge of the clinical trials and contributed to interpretations of the data; RK, OP and MW participated in designing the investigation and gave interpretations of the data. All authors read and approved the final manuscript.
